# Geriatric Emergency Care Reduces Health Care Costs—What Are the Next Steps?

**DOI:** 10.1001/jamanetworkopen.2021.0147

**Published:** 2021-03-01

**Authors:** Maura Kennedy, Kei Ouchi, Kevin Biese

**Affiliations:** Department of Emergency Medicine, Massachusetts General Hospital, Boston; Department of Emergency Medicine, Harvard Medical School, Boston, Massachusetts; Department of Emergency Medicine, Harvard Medical School, Boston, Massachusetts; Department of Emergency Medicine, Brigham and Women’s Hospital, Boston, Massachusetts; Department of Emergency Medicine, University of North Carolina at Chapel Hill; West Health, La Jolla, California

Although older adults frequently receive care in emergency departments (EDs), conventional EDs may not adequately address the unique needs of geriatric patients, such as managing geriatric syndromes, addressing multimorbidity, and optimizing care transitions.^1^ In direct response to the unique medical needs of older patients, the first self-identified geriatric ED (GED) in the United States was established more than a decade ago, after which there has been a rapid increase in the number of GEDs.^1^ In 2018, the American College of Emergency Physicians launched a voluntary accreditation program, classifying GEDs as level 1 (gold), level 2 (silver), or level 3 (bronze) based on staffing, care processes, physical environment, and specialized equipment.^2^ Despite rapid growth in the number of GEDs in the United States, there is limited research on the impact of GEDs and specialized geriatric emergency care models.

The most robust evidence supporting the GED model of care comes from the Geriatric Emergency Department Innovation in Care Through Workforce, Informatics, and Structural Enhancement (GEDI WISE) program. This multicenter care innovation program was supported by a Centers for Medicare & Medicaid Services (CMS) Health Care Innovations Award. It includes transitional care nurses (TCNs) and social workers (SWs) who staff the GEDI WISE level 1 GEDs and conduct geriatric assessments (including evaluations for delirium, fall risk, and functional decline), engage in conversations about goals of care, and assist with care coordination.^3^ To date, the GEDI WISE investigators have demonstrated that this care model can decrease hospital admissions, future ED visits, and 30-day hospital readmissions.^4,5^

Elsewhere in *JAMA Network Open*, Hwang et al^3^ measured the cost savings associated with TCN or SW evaluations at GEDI WISE sites. Using entropy balancing, they compared the total Medicare costs for 30 and 60 days after the index ED visit among an intervention group of fee-for-service Medicare beneficiaries who received a TCN or SW evaluation and a control group of fee-for-service Medicare beneficiaries who did not. Whereas the prior GEDI WISE studies evaluated the association of this care model with ED visits and hospital admission rates, in this study Hwang et al^3^ conducted a more comprehensive evaluation of costs of care by looking at total Medicare costs—including inpatient, outpatient, home health, hospice, and skilled nursing facilities claims. In doing so, they not only quantified the cost savings related to avoidance of hospitalization and ED revisits but also determined whether the cost savings were offset by increased care costs related to home health referrals or transfer to skilled nursing or acute rehabilitation facilities. Hwang et al^3^ found that after entropy balancing, patients who received the TCN or SW intervention had a mean 30-day savings of $2436 per beneficiary at 1 site and $2905 per beneficiary at the other site. The cost savings persisted at 60 days, with a mean savings of $1200 at 1 site and $3202 at the second site.

This study provides important evidence demonstrating the cost efficiency of this enhanced care model for geriatric emergency care. Evidence that higher-quality models of care, such as GEDs, can reduce health care costs should catalyze the adoption of these models. This is more likely to happen when the savings generated benefit the entity shouldering the costs. However, in the GEDI WISE program, the savings went to the payer, in this case Medicare, while the costs of sustaining this intervention beyond the grant-funded period were borne by the hospitals. Asking hospitals to spend their own money to save Medicare money is unlikely to be sustainable. Growth of this care model requires that health care systems also benefit financially from the cost savings.

The traditional way Medicare has incentivized individual clinicans, hospitals, and health care systems to adopt new services is to directly pay for it—allowing clinicians and health care systems to submit bills for the services they have provided. While this is a mechanism that could be used to reimburse facilities for a TCN or SW consultation or a similar GED care model, creating new billing codes is an arduous and prolonged process and not likely to create additional revenue for GEDs anytime soon. CMS is encouraging the adoption of value-based care models in which organizations (health care systems and clinician networks) financially benefit from providing high-value care. CMS is accomplishing this by encouraging organizations to take on financial risk for managing patient populations in risk-sharing Accountable Care Organization (ACO) models. These models hold organizations responsible for financial risk if they provide low-value (ie, expensive and/or low-quality) care to their patient populations.^6^ In these advanced ACO reimbursement models, the organization receives additional revenue when savings are generated and quality of care is maintained or improved. Another mechanism for funding advanced geriatric emergency care models is through collaboration with Medicare Advantage plans, given that these plans benefit from reducing costs while improving quality of care. Health care systems should collaborate with ACOs and/or their local Medicare Advantage plans when implementing a GED model of care because these parties all stand to benefit from the savings demonstrated in this study.

Home-based care alternatives are another option that is cost saving for payers and revenue generating for hospitals. Instead of admitting an older adult with complex care needs to the hospital, a GED could admit that patient to a hospital-at-home program or leverage telehealth and/or remote monitoring programs for follow-up. Telehealth may also be a viable solution for disseminating GED models of care across the United States. While the GEDI WISE sites are level 1 GEDs in large metropolitan areas, at least 18% of older adults reside in rural areas.^7^ We must consider how geriatric care models developed in large academic EDs in metropolitan areas can be disseminated to small EDs in rural locales. Telehealth is an obvious answer. Through a telehealth platform, a single TCN or SW could conduct consultations across multiple EDs, improving efficiency and expanding access to this care model.

The next phase of research into models for geriatric emergency care must examine the association of GEDs with carefully selected, patient-centered outcomes that matter most to older adults. This research may include evaluation of GED care models on physical functioning, cognition, and quality of life. For instance, does avoidance of hospitalization through the GED model of care result in improved physical activity and prevent functional decline? Will GED models of care prevent delirium in older adults with acute medical care needs? Do GED initiatives improve quality of life in older adults requiring emergency care? Quality of life is a measure that encompasses an individual’s physical, mental, emotional, and social functioning and could serve as a composite patient-oriented outcome in future studies evaluating the consequences of geriatric emergency care innovations.

Now that the cost savings of enhanced geriatric emergency care are clear, we need to consider how we can leverage payer models to increase adoption of these programs and disseminate them to nonmetropolitan areas. We also need to conduct robust research to evaluate the association of GEDs with outcomes among older adults using a patient-centered approach and focusing on what matters most to this population. Through this framework, we can increase access to high-quality, cost-effective, geriatric-centric emergency care in the United States for older adults.

## References

[1] Schumacher JG, Hirshon JM, Magidson P, Chrisman M, Hogan T. Tracking the rise of geriatric emergency departments in the United States. J Appl Gerontol. 2020;39(8):871–879. doi:10.1177/0733464818813030 30451060

[2] American College of Emergency Physicians. ACEP launches geriatric emergency department accreditation program. Published 5 10, 2018. Accessed October 26, 2020. https://www.acep.org/globalassets/sites/acep/media/geda-documents/gedapilotannoucement.pdf

[3] Hwang U, Dresden SM, Vargas-Torres C, ; Geriatric Emergency Department Innovations in Care Through Workforce, Informatics, and Structural Enhancement (GEDI WISE) Investigators. Association of a geriatric emergency department innovation program with cost outcomes among Medicare beneficiaries. JAMA Netw Open. 2021;4(3):2037334. doi:10.1001/jamanetworkopen.2020.37334 PMC7921898 33646311

[4] Hwang U, Dresden SM, Rosenberg MS, ; GEDI WISE Investigators. Geriatric emergency department innovations: transitional care nurses and hospital use. J Am Geriatr Soc. 2018;66(3):459–466. doi:10.1111/jgs.15235 29318583 PMC6764445

[5] Dresden SM, Hwang U, Garrido MM, Geriatric emergency department innovations: the impact of transitional care nurses on 30-day readmissions for older adults. Acad Emerg Med. 2020;27(1):43–53. doi:10.1111/acem.13880 31663245 PMC13261736

[6] Peck KA, Usadi B, Mainor AJ, Fisher ES, Colla CH. ACO contracts with downside financial risk growing, but still in the minority. Health Aff (Millwood). 2019;38(7):1201–1206. doi:10.1377/hlthaff.2018.05386 31260361 PMC8559781

[7] Smith AS, Trevelyan E. The older population in rural America: 2012-2016. Published 9 2019. Accessed December 4, 2020. https://www.census.gov/content/dam/Census/library/publications/2019/acs/acs-41.pdf

